# Study on the expression of TRIM7 in peripheral blood mononuclear cells of patients with sepsis and its early diagnostic value

**DOI:** 10.1186/s12879-022-07874-6

**Published:** 2022-11-19

**Authors:** Mingfeng Lu, Aiwen Ma, Jianwei Liu, Wenzhen Zhou, Peng Cao, Tao Chu, Lu Fan

**Affiliations:** Department of Emergency, Clinical Medical College, Yangzhou University, No 98, Nantong West Rd, Yangzhou, 225001 China

**Keywords:** TRIM7, PBMCs, Sepsis, Diagnosis

## Abstract

**Background:**

The early diagnosis of sepsis is beneficial to put forward a reasonable clinical treatment plan as soon as possible. This study was to explore the expression of Tripartite Motif 7 (TRIM7) in peripheral blood mononuclear cells (PBMCs) of patients with sepsis and its diagnostic value.

**Methods:**

This is a cross-sectional study. A total of 69 patients with infectious diseases were enrolled in the emergency room. They were divided into the sepsis group (34 cases) and the non-sepsis infection group (35 cases). There were 25 healthy subjects who were selected as the control group. The expression of TRIM7 in PBMCs was observed by immunofluorescence staining. The correlation between the expression of TRIM7 mRNA and acute physiology and chronic health evaluation II (APACHE II) score, sequential organ failure assessment (SOFA) score, white blood cell (WBC), C-reactive protein (CRP), procalcitonin (PCT), tumor necrosis factor (TNF)-α and interleukin (IL)-6 was discussed. The receiver operating characteristic (ROC) curve was utilized for evaluating the value of TRIM7 expression for the early diagnosis of sepsis.

**Results:**

The fluorescence intensity representing the expression level of TRIM7 in PBMCs of patients in the sepsis group was the lowest among three groups. The TRIM7 mRNA expression in PBMCs of the sepsis group was greatly decreased in comparison with that of the non-sepsis infection group and control group (*P* < 0.05). Spearman correlation analysis indicated that TRIM7 mRNA expression was negatively correlated with APACHE II score, SOFA score, WBC, CRP, PCT, TNF-α and IL-6. ROC curve analysis revealed that the area under curve (AUC) of TRIM7 mRNA expression in PBMCs for the diagnosis of sepsis was 0.798, with a 95% confidence interval of 0.691- 0.905, a sensitivity of 73.5%, and a specificity of 77.1%.

**Conclusion:**

The expression of TRIM7 in PBMCs of patients with sepsis is significantly down-regulated, which has certain clinical value for early diagnosis of sepsis.

## Background

Sepsis is a life-threatening organ dysfunction caused by uncontrolled response to infection [1]. Annually, more than 19 million sepsis cases are reported in the world, of which about 6 million patients die, and about 3 million people survive with different degrees of dysfunction [2–4]. Early identification of whether patients with infection may progress to sepsis or even septic shock is very important for guiding clinical treatment [5]. Commonly used indicators for evaluating the severity of infection and inflammation include white blood cell (WBC), C-reactive protein (CRP), procalcitonin (PCT) [6], tumor necrosis factor (TNF)-α and interleukin (IL)-6 [7], etc. Clinical studies have shown that the above indicators have some defects in sensitivity or specificity. As a supplement to these indicators, some new markers may have positive significance in the prediction and early diagnosis of sepsis.

Recently, increasing studies have reported proteins with a role in the regulation of inflammation mediated by the Toll-like receptor 4 (TLR4) signaling pathway. The variations in the expression level of these proteins reflect the degree of the body’s inflammatory response to some extent [8, 9]. TRIM7 protein belongs to the E3 ubiquitin ligase family. Studies have found that by activating the TLR4 signaling pathway, TRIM7 can promote the release of inflammatory factors under the action of infectious factors, and its expression level is down-regulated in mouse peritoneal macrophages stimulated by LPS [10]. This study detected the expression of TRIM7 in peripheral blood mononuclear cells (PBMCs) of patients with sepsis at an early stage and analyzed its value in the early diagnosis of sepsis.

## Methods

### Research design

In this cross-sectional study, a total of 34 patients with sepsis (sepsis group) and 35 patients with non-septic infectious diseases (non-septic infection group) were enrolled from the emergency room of Northern Jiangsu People’s Hospital from January to December 2020. Twenty-five healthy people were selected as the control group. The patients with sepsis met the sepsis 3.0 diagnostic criteria issued by the Society of Critical Care Medicine (SCCM) and the European Society of Intensive Care Medicine (ESICM) in 2016 [1].

### Inclusion and exclusion criteria

The inclusion criteria of sepsis group and non-septic infection group: ① Age > 18 years; ② The sepsis group met the diagnostic criteria of sepsis [1]; ③ Newly diagnosed patients who were untreated or treated with antibiotics less than 48 h [11]; ④ Patients with complete clinical data. The exclusion criteria: ① Patients with autoimmune diseases; ② Patients with potential immunosuppression (such as AIDS, malignant tumors under treatment, organ or bone marrow transplantation, drug-induced leucopenia); ③ Pregnant women. The inclusion criteria for the control group: ① Age > 18 years; Exclusion criteria: ① Patients who had bacterial or viral infection within 1 month before enrollment; ② Patients who were treated with antibiotics within 1 month before enrollment.

### Collection of clinical data

The baseline clinical data at admission were recorded, including age, sex, comorbid conditions, and infection site. Acute physiology and chronic health evaluation II (APACHE II) and sequential organ failure assessment (SOFA) scores were performed on all subjects. On the day of admission, a blood routine examination was performed by the automatic analyzer, WBC count was recorded, CRP concentration in plasma was detected by rate turbidimetry, and procalcitonin (PCT) level was detected by chemiluminescence.

### Detection of TNF-α and IL-6 in plasma

The concentration of TNF-α in plasma was detected by the human cytokine ELISA kit (ehc103a.96, NeoBioscience), and the concentration of IL-6 in plasma was detected by the human cytokine ELISA kit (ehc007.96, NeoBioscience). All samples were measured twice to obtain the mean value.

### The expression of TRIM7 in PBMCs

#### The separation of PBMCs

When patients were admitted to the hospital, a total of 5mL venous blood was drawn and placed into anticoagulation tubes. PBMCs were extracted from the blood sample by gradient centrifugation with Ficoll Paque (71101900-EH, GE Healthcare Bio-Sciences). The cells were re-suspended in PBS solution for immunofluorescence and Real-time Quantitative PCR (qPCR) detection of TRIM7.

#### The expression of TRIM7 protein in PBMCs detected by immunofluorescence

The concentration of PBMCs suspension was adjusted to 5 × 10^6^/mL with PBS, and 20 µL of PBMCs suspension for each sample was dripped onto glass slides, fixed with 4% paraformaldehyde, and sealed with 0.1% TritonX-100 and 1% bovine serum albumin (BSA). Rabbit polyclonal antibody to TRIM7 (LS-C146201, LifeSpan Biosciences) was used for the primary antibody, Goat anti-Rabbit IgG-FITC antibody (HA1004, Hangzhou HuaAn Biotechnology) was used for the secondary antibody, and 4′,6-diamidino-2-phenylindole (DAPI) staining solution (C1002, Beyotime) was used for staining cell nucleus. After sealing by Prolong, the slides were observed and photographed under a laser confocal fluorescence microscope (LSM710, Zeiss). We used ImageJ software to analyze the brightness of the cells in the immunofluorescence maps, and randomly selected 20 cells in each group for quantitative detection and took their mean values for comparison.

#### Detection of the expression level of TRIM7 mRNA

The TRIM7 mRNA in PBMCs was isolated from the same sample for the fluorescence experiment of TRIM7 protein. RNA was extracted from PBMCs with the RNA extraction kit (CW0584S, cwbio) and eluted with 30 µL elusion buffer, which is dedicated for RNA extraction. To prevent RNA degradation, the RNA extraction experiment was carried out immediately after separation of PBMCs. All equipment including pipetting tips, EP tubes and experimental reagents were RNase-free. The gloves were changed in time during the experiment. cDNA was produced from RNA through reverse transcription. Real-time PCR was performed on Roche Light Cycler 2.0 fluorescent quantitative PCR instrument by employing the SYBR Green Real-time PCR Master Mix kit (E096-01A, Novoprotein). The data were processed with Light Cycle Software 4.1, and the Ct value of the target genes was standardized with the Ct value of human *GAPDH*. The 2^−△△Ct^ method was used to calculate the variation of the amplification multiple of the sample. The quantitative primer sequences were as follows:

- *GAPDH* sense 5′GGAGCGAGATCCCTCCAAAAT 3′,
- *GAPDH* antisense 5′GGCTGTTGTCATACTTCTCATGG 3′;
- *TRIM7 *sense 5′TCCATGTTCAAGTCCCTCTCC 3′,
- *TRIM7 *antisense 5′GGCCAGGTTCTCATTCTGCT 3′.

### Statistical methods

Statistical Product and Service Solutions (SPSS) 26.0 was utilized for statistical analysis. Continuous variables with normal distribution were presented as mean ± SD. Variables with abnormal distribution were presented as median (Inter quartile range, IQR). ANOVA or Kruskal Wallis was employed for analyzing the comparison among the three groups. The chi-square test or Fisher’s exact test was used to compare categorical variables. The spearman analysis method was used for correlation analysis. The ROC curve was applied for evaluating the early diagnostic value of TRIM7 expression in PBMCs in sepsis. All statistics were two-sided, only a *p*-value of 0.05 or less was considered statistically significant.

## Results

### Demographic characteristics and clinical parameters of patients in each group

In this study, 34 patients in sepsis group, 35 patients in non-septic infection group and 25 healthy controls were collected. The pathogens of all patients infected in the experiment were considered as bacteria. There was no significant difference in age, sex, comorbid conditions, or infection site among the three groups (*p* > 0.05). Organ function scores including APACHE II, SOFA scores, and inflammatory indexes including CRP, PCT, TNF-α and IL-6 were significantly different among the three groups. These parameters of the sepsis group were considerably elevated in comparison with those of the non-septic infection group and control group (*p* < 0.05); WBC levels in the sepsis group and non-septic infection group were higher than those in control group (*p* < 0.05), but no notable difference was observed between the sepsis group and the non-septic infection group (Table 1).

**Table 1:** Demographic characteristics, organ function scores, inflammatory indexes and TRIM7 mRNA level of the subjects in three groups

Characteristic	Health control	Non-septic infection	Sepsis	*p* value
Demographics				
N	25	35	34	
Age (years)	63.3 ± 14.67	68.6 ± 14.22	68.2 ± 13.14	0.297
Gender, male (%)	14	15	20	0.374
Comorbid conditions N (%)				
Diabetes mellitus	6	10	12	0.632
Cardiac insufficiency	5	15	15	0.114
Chronic obstructive pulmonary disease	1	4	3	0.594
Chronic renal insufficency	2	2	3	0.879
Chronic liver disease	1	2	3	0.740
Malignancy	3	1	5	0.220
Site of infection N (%)				
Respiratory		10	15	0.209
Urinary		9	8	0.833
Abdominal		7	8	0.722
Musculoskeletal, skin and soft tissue		5	5	0.950
Bloodstream		7	6	0.803
Others		3	1	0.317
Organ function indices and inflammatory biomarkers				
APACHE II	5 (2, 6)	9 (7, 12)^a^	20 (14, 25)^a,b^	0.000
SOFA	0 (0, 0)	2 (1, 3)^a^	7 (6, 10)^a,b^	0.000
WBC (10^9^/L)	6.7 ± 2.72	13.7 ± 4.49^a^	14.2 ± 6.77^a^	0.000
CRP (mg/L)	2.6 ± 3.14	64.2 ± 42.37^a^	114.0 ± 78.10^a,b^	0.000
PCT (µg/L)	0.16 (0.05, 0.26)	1.19 (0.12, 8.21)^a^	10.10 (3.80, 62.36)^a,b^	0.000
TNF-α (ng/L)	24.34 (19.17, 43.09)	36.98 (25.12, 68.23)^a^	67.48 (28.95, 92.29)^a,b^	0.000
IL-6 (ng/L)	13.30 (6.90, 35.64)	43.05 (23.45, 81.90)^a^	115.33 (60.78, 246.31)^a,b^	0.000
TRIM7 mRNA (2^−△△Ct^)	8.06 (5.28, 13.38)	2.86 (1.88, 3.97)^a^	1.10 (0.83, 2.00)^a,b^	0.000

Continuous variables with normal distribution were presented as mean ± SD. Variables with abnormal distribution were presented as median (IQR). ANOVA or Kruskal Wallis was employed for analyzing the comparison among the three groups. The chi-square test or Fisher’s exact test was used to compare categorical variables

*APACHE II * acute physiology and chronic health evaluation II, *SOFA* sequential organ failure assessment, *WBC* white blood cell, *CRP* C-reactive protein, *PCT* procalcitonin, *TNF-α* tumor necrosis factor-α, *IL-6* interleukin-6

^a^Compared to control group

^b^Compared to non-septic group

### The expression level of TRIM7 protein in PBMCs of each group detected by immunofluorescence

**Fig. 1:**
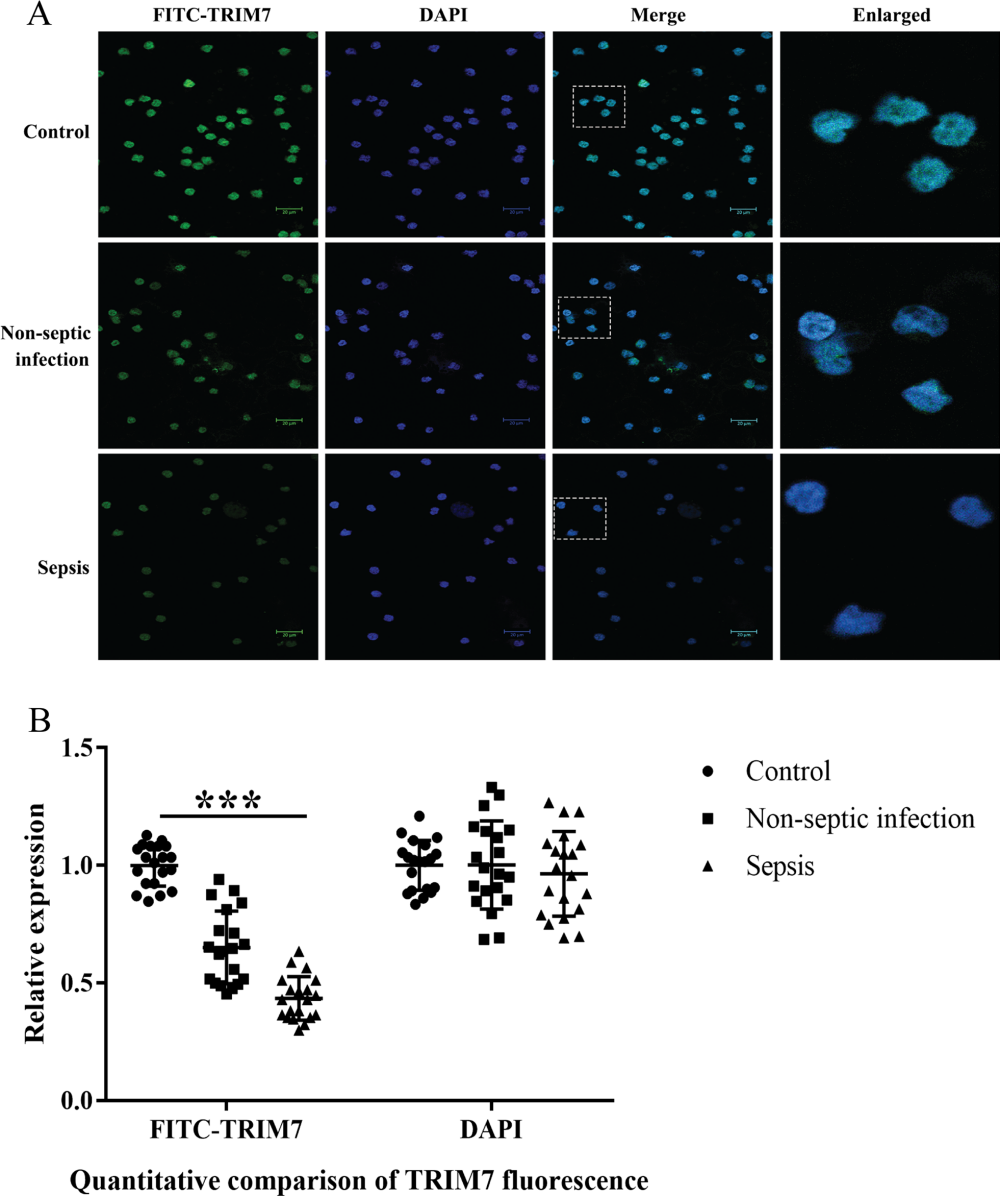
The expression level of TRIM7 protein in PBMCs of each group detected by immunofluorescence. **A** Immunofluorescence images showing staining of PBMCs of individuals from sepsis group, non-septic infection group and health control group with DAPI (blue), or antibodies against TRIM7 (green). Images were taken at the magnification of 400. Scale bar represents 20 μm. **B** The quantitative analysis with ImageJ software revealed that the expression level of TRIM7 in PBMCs of patients in the sepsis group was the lowest among three groups. The fluorescence intensity of TRIM7 in non-septic infection group was also down-regulated than that of the control group. The expression level of DAPI in PBMCs among the three groups showed no significant difference.

DAPI was used to label the nucleus (blue), fluorescein isothiocyanate (FITC) was used to label TRIM7 protein (green), and a laser confocal microscope was employed for fluorescence detection. The outcomes revealed that the positive TRIM7 protein expression was mainly located in the nucleus, showing green fluorescence. The green fluorescence intensity representing the expression level of TRIM7 in PBMCs of patients in the sepsis group was the lowest among three groups. The fluorescence intensity of TRIM7 in non-septic infection group was also down-regulated than that of the control group (Fig. 1).

### The expression level of* TRIM7* mRNA in PBMCs of each group and its correlation with inflammatory indexes and organ function scores

The expression of *TRIM7* mRNA in PBMCs of the non-septic infection group was substantially down-regulated than that of control group (*p* < 0.05). The expression of *TRIM7* mRNA in PBMCs of the sepsis group was significantly reduced to the lowest level comparing to that of non-septic infection group and control group (*p* < 0.05) (Table 1). Spearman correlation analysis highlighted that the expression level of *TRIM7 *mRNA in PBMCs was negatively associated with APACHE II score, SOFA score, WBC, CRP, PCT, TNF-α, and IL-6 (Table 2).

**Table 2:** The correlation between *TRIM7* mRNA expression and organ function scores, inflammatory indexes

Related indexes	APACHE II	SOFA	WBC (10^9^/L)	CRP (mg/L)	PCT (ng/L)	TNF-α (ng/L)	IL-6 (ng/L)
r_s_	− 0.699	− 0.690	− 0.336	− 0.516	− 0.584	− 0.318	− 0.454
*p* value	0.000	0.000	0.001	0.000	0.000	0.002	0.000

The spearman analysis method was used for correlation analysis

*APACHE II * acute physiology and chronic health evaluation II, *SOFA* sequential organ failure assessment, *WBC* white blood cell, *CRP* C-reactive protein, *PCT* procalcitonin, *TNF-α* tumor necrosis factor-α, *IL-6* interleukin-6

### Diagnostic value of the expression level of TRIM7 in PBMCs for sepsis

**Fig. 2:**
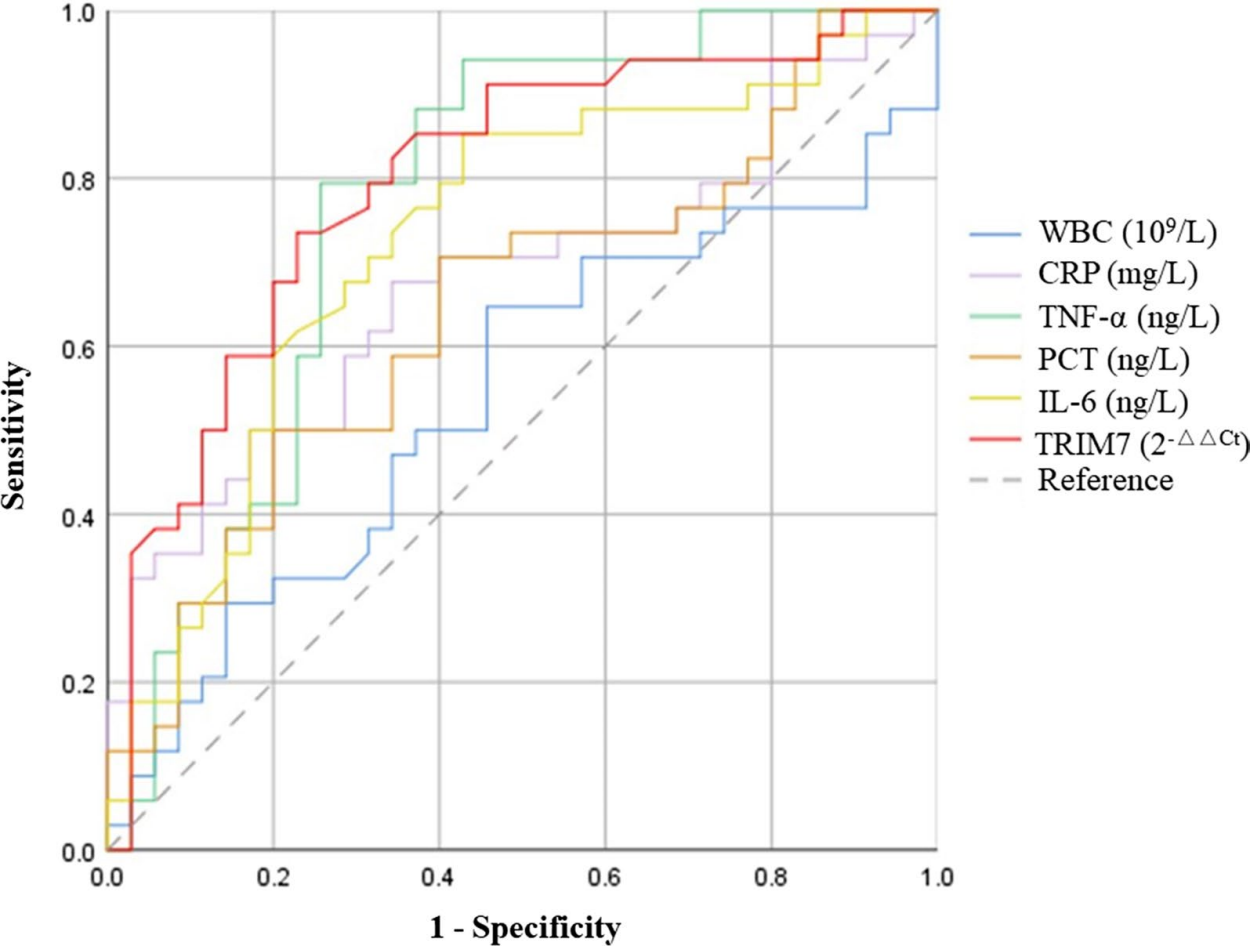
ROC curves of the *TRIM7* mRNA level in PBMCs and clinical indexes for sepsis diagnosis

ROC curve analysis revealed that the AUC of *TRIM7* mRNA expression in PBMCs for the diagnosis of sepsis was 0.798, with a 95% confidence interval of 0.691-0.905, a sensitivity of 73.5%, and a specificity of 77.1% (Table 3). Figure 2 showed the comparison of the diagnostic value for sepsis between *TRIM7* and other commonly used clinical indicators.

**Table 3:** Areas under the curve of *TRIM7* mRNA level and inflammatory parameters for determining sepsis

Parameter	Cut-off value	AUC	Sensitivity	Specifity	*p* value	95% CI	
						Lower limit	Higher limit
WBC (10^9^/L)	14.105	0.541	0.647	0.543	0.560	0.401	0.680
CRP (mg/L)	74.955	0.676	0.676	0.657	0.012	0.547	0.805
PCT (µg/L)	3.680	0.772	0.794	0.743	0.000	0.658	0.887
TNF-α (ng/L)	49.512	0.647	0.706	0.600	0.036	0.516	0.778
IL-6 (ng/L)	47.250	0.729	0.853	0.571	0.001	0.608	0.850
*TRIM7* (2^−△△Ct^)	1.829	0.798	0.735	0.771	0.000	0.691	0.905

The ROC curve was applied for evaluating the early diagnostic value of *TRIM7* expression and inflammatory parameters in PBMCs in sepsis

*AUC* area under curve, *CI* confidence interval, *WBC* white blood cell, *CRP* C-reactive protein, *PCT* procalcitonin, *TNF-α* tumor necrosis factor-α, *IL-6* interleukin-6

## Discussion

With the rapid development of molecular biology and laboratory testing technology, increasing researches have been devoted to explore the new biomarkers in diagnosis and severity evaluation for sepsis [12]. Toll-like receptors (TLRs) play an important role in sepsis [13], among which TLR4 has been widely studied and considered as a key molecule in the innate immune system, participating in the occurrence and development of sepsis [14]. TLR4 is a crucial transmembrane receptor that can mediate both the inflammatory response to exogenous ligands like lipopolysaccharide (LPS) and the endogenous danger signals generated at the time of inflammatory response [15]. In LPS-induced sepsis, activation of TLR4 causes downstream NF-κB and MAPKs pathways to be activated in a MyD88-dependent manner, thereby enhancing pro-inflammatory cytokines including TNF-α and IL-6 [16]. Many proteins that regulate the TLR4 pathway have come to the attention of researchers. On one hand, the expression level of these protein molecules is related to the TLR4-activated inflammatory response, which can be used to evaluate the severity of the infection and predict the occurrence of sepsis [17–19]. On the other hand, these protein molecules may become potential targets for the early diagnosis and treatment of sepsis [20, 21].

As an E3 ubiquitin ligase, TRIM7 participates in some important biological processes, such as glycogen metabolism and tumor cell proliferation [22, 23]. Studies have suggested that TRIM7 may play an antiviral role against norovirus [24]. Our previous research also found that TRIM7 can participate in the regulation of innate immunity through the TLR4 signaling pathway, which positively regulates the activation of downstream NF-κB and MAPKs pathways. Our previous cell experiments showed that the expression level of TRIM7 was down-regulated immediately in mouse peritoneal macrophages after LPS stimulation, then recovered to the initial level after 24 h [10]. According to the above research results, the expression level of TRIM7 may reflect the severity of infectious diseases and may predict the occurrence of sepsis in the early stage of infection.

PBMCs can contact with pathogens and interact with infected cells through secreted signal molecules. They have a recognized role in monitoring infection [25]. For example, Zhang et al. discovered that the decreased expression of miR-23b in PBMCs of patients with sepsis was negatively correlated with the inflammatory response, which could reduce the release of inflammatory cytokines stimulated by LPS [26]. Our research found that the expression level of TRIM7 in PBMCs of patients with non-septic-infection was lower than that of normal controls, while the expression level of TRIM7 in PBMCs of patients with sepsis was lower than that of patients with non-septic-infection. At the same time, the expression of TRIM7 was negatively correlated with disease severity scores including SOFA [27], APACHE II score [28], and inflammation-related indicators including WBC, CRP, PCT, TNF-α, and IL-6. The expression of TRIM7 in PBMCs of individuals with infection is closely related to its biological role in affecting inflammatory response through regulating the TLR4 pathway, and its change trend is consistent with the results of our previous cell experiments. The outcomes of the present research revealed that the reduced level of TRIM7 expression in PBMCs may indicate a more serious condition in infectious diseases. Intriguingly, our previous experiments have confirmed that overexpression of TRIM7 can activate NF-κB and MAPKs pathways, resulting in increased release of TNF-α, IL-6 and other inflammatory factors, thus aggravating the inflammatory response. Knockdown of TRIM7 can reduce the inflammatory response, suggesting that TRIM7 can positively regulate the inflammatory response after infection [10]. We assume that the down-regulation of TRIM7 expression in PBMCs of sepsis patients may be related to the host’s self-defense after infection. The mechanism of the above phenomenon deserves further research.

Early identification and diagnosis of sepsis are conducive to the rapid development of a reasonable treatment plan, which is of great value in improving the prognosis of patients with sepsis. In the present research, we also assessed the diagnostic values of commonly used clinical indicators for sepsis. ROC curve analysis revealed that the diagnostic value of plasma WBC, CRP, and TNF-α for sepsis was dissatisfactory, while PCT and IL-6 were slightly better. The AUC of *TRIM7* mRNA’s level in PBMCs for the diagnosis of sepsis was 0.798, the sensitivity was 73.5%, and the specificity was 77.1%. As an early diagnostic index, it shows a good sensitivity while its specificity even exceeds PCT, which is commonly used in clinic. The good specificity may be attributed to that the detection object of TRIM7 expression is PBMCs, which directly participates in the immune reaction after pathogen infection [24]. In addition, our prior research also verified that TRIM7 can regulate the expression of inflammatory factors after infection through TLR4 pathway, which makes it an important molecular in the development of sepsis [10]. By comparing with other clinical indicators, we can see that *TRIM7* mRNA level in PBMCs has certain advantages in the early diagnosis of sepsis.

## Conclusion

In conclusion, the expression level of TRIM7 in PBMCs of patients with sepsis is significantly down-regulated, which has a certain value for early diagnosis of sepsis and can be used as a supplement to common infection markers in clinical work. Since the change of TRIM7 expression in cell experiments mainly occurs in the early stage of infection [10], early and dynamic monitoring is required to accurately evaluate the severity of infectious diseases and predict the development of sepsis. There are some limitations in this study. We have enrolled a small size of population, and only recorded the TRIM7 expression of patients in the early stage of admission. In the future, the sample size can be expanded and the patients can be dynamically monitored to verify the existing research results.

## Data Availability

All data generated or analyzed during this study were included in this manuscript.

## References

[1] Singer M, Deutschman CS, Seymour CW, Shankar-Hari M, Annane D, Bauer M, Bellomo R, Bernard GR, Chiche JD, Coopersmith CM, Hotchkiss RS, Levy MM, Marshall JC, Martin GS, Opal SM, Rubenfeld GD, van der Poll T, Vincent JL, Angus DC (2016). The third international consensus definitions for sepsis and septic shock (Sepsis-3). JAMA.

[2] Perner A, Cecconi M, Cronhjort M, Darmon M, Jakob SM, Pettilä V, van der Horst ICC (2018). Expert statement for the management of hypovolemia in sepsis. Intensive Care Med.

[3] Prescott HC, Angus DC (2018). Post sepsis morbidity. JAMA.

[4] Reinhart K, Daniels R, Kissoon N, Machado FR, Schachter RD, Finfer S (2017). Recognizing sepsis as a global health priority—a WHO resolution. N Engl J Med.

[5] Qu Z, Zhu Y, Wang M, Li W, Zhu B, Jiang L, Xi X (2021). Prognosis and risk factors of sepsis patients in Chinese ICUs: a retrospective analysis of a cohort database. Shock.

[6] De Oro N, Gauthreaux ME, Lamoureux J, Scott J (2019). The use of procalcitonin as a sepsis marker in a community hospital. J Appl Lab Med.

[7] Kobayashi T, Iwatani S, Hirata A, Yamamoto M, Yoshimoto S (2021). Rapid changes in serum IL-6 levels in preterm newborns with Gram-negative early-onset sepsis. Cytokine.

[8] Ebner P, Versteeg GA, Ikeda F (2017). Ubiquitin enzymes in the regulation of immune responses. Crit Rev Biochem Mol Biol.

[9] Wang Q, Huang L, Hong Z, Lv Z, Mao Z, Tang Y, Kong X, Li S, Cui Y, Liu H, Zhang L, Zhang X, Jiang L, Wang C, Zhou Q (2017). The E3 ubiquitin ligase RNF185 facilitates the cGAS-mediated innate immune response. PLoS Pathog.

[10] Lu M, Zhu X, Yang Z, Zhang W, Sun Z, Ji Q, Chen X, Zhu J, Wang C, Nie S (2019). E3 ubiquitin ligase tripartite motif 7 positively regulates the TLR4-mediated immune response via its E3 ligase domain in macrophages. Mol Immunol.

[11] Winiszewski H, Despres C, Puyraveau M, Lagoutte-Renosi J, Montange D, Besch G, Floury SP, Chaignat C, Labro G, Vettoretti L, Clairet AL, Capellier G, Vivet B, Piton G (2022). β-Lactam dosing at the early phase of sepsis: performance of a pragmatic protocol for target concentration achievement in a prospective cohort study. J Crit Care.

[12] Wang H, Huang J, Yi W, Li J, He N, Kang L, He Z, Chen C (2022). Identification of immune-related key genes as potential diagnostic biomarkers of sepsis in children. J Inflamm Res.

[13] Li HR, Liu J, Zhang SL, Luo T, Wu F, Dong JH, Guo YJ, Zhao L (2017). Corilagin ameliorates the extreme inflammatory status in sepsis through TLR4 signaling pathways. BMC Complement Altern Med.

[14] Chantratita N, Tandhavanant S, Seal S, Wikraiphat C, Wongsuvan G, Ariyaprasert P, Suntornsut P, Teerawattanasook N, Jutrakul Y, Srisurat N, Chaimanee P, Mahavanakul W, Srisamang P, Phiphitaporn S, Mokchai M, Anukunananchai J, Wongratanacheewin S, Chetchotisakd P, Emond MJ, Peacock SJ, West TE (2017). TLR4 genetic variation is associated with inflammatory responses in Gram-positive sepsis. Clin Microbiol Infect.

[15] Park BS, Lee JO (2013). Recognition of lipopolysaccharide pattern by TLR4 complexes. Exp Mol Med.

[16] Wang Z, Chen W, Li Y, Zhang S, Lou H, Lu X, Fan X (2021). Reduning injection and its effective constituent luteoloside protect against sepsis partly via inhibition of HMGB1/TLR4/NF-κB/MAPKs signaling pathways. J Ethnopharmacol.

[17] Na L, Ding H, Xing E, Zhang Y, Gao J, Liu B, Yu J, Zhao Y (2020). The predictive value of microRNA-21 for sepsis risk and its correlation with disease severity, systemic inflammation, and 28-day mortality in sepsis patients. J Clin Lab Anal.

[18] Xie Z, Guo Z, Liu J (2018). Whey acidic protein/four-disulfide core domain 21 regulate sepsis pathogenesis in a mouse model and a macrophage cell line via the Stat3/Toll-Like receptor 4 (TLR4) signaling pathway. Med Sci Monit.

[19] Kuzmich NN, Sivak KV, Chubarev VN, Porozov YB, Savateeva-Lyubimova TN, Peri F (2017). TLR4 signaling pathway modulators as potential therapeutics in inflammation and sepsis. Vaccines (Basel).

[20] Loubaki L, Chabot D, Paré I, Drouin M, Bazin R (2017). MiR-146a potentially promotes IVIg-mediated inhibition of TLR4 signaling in LPS-activated human monocytes. Immunol Lett.

[21] Venancio TM, Machado RM, Castoldi A, Amano MT, Nunes VS, Quintao EC, Camara NO, Soriano FG, Cazita PM (2016). CETP lowers TLR4 expression which attenuates the inflammatory response induced by LPS and polymicrobial sepsis. Mediat Inflamm.

[22] Montori-Grau M, Pedreira-Casahuga R, Boyer-Díaz Z, Lassot I, García-Martínez C, Orozco A, Cebrià J, Osorio-Conles O, Chacón MR, Vendrell J, Vázquez-Carrera M, Desagher S, Jiménez-Chillarón JC (2018). Gómez-Foix AM. GNIP1 E3 ubiquitin ligase is a novel player in regulating glycogen metabolism in skeletal muscle. Metabolism.

[23] Zhu L, Qin C, Li T, Ma X, Qiu Y, Lin Y, Ma D, Qin Z, Sun C, Shen X, Zhao Y, Han L (2020). The E3 ubiquitin ligase TRIM7 suppressed hepatocellular carcinoma progression by directly targeting src protein. Cell Death Differ.

[24] Orchard RC, Sullender ME, Dunlap BF, Balce DR, Doench JG, Virgin HW (2018). Identification of Antinorovirus genes in human cells using genome-wide CRISPR activation screening. J Virol.

[25] Boldrick JC, Alizadeh AA, Diehn M, Dudoit S, Liu CL, Belcher CE, Botstein D, Staudt LM, Brown PO, Relman DA (2002). Stereotyped and specific gene expression programs in human innate immune responses to bacteria. Proc Natl Acad Sci USA.

[26] Zhang W, Lu F, Xie Y, Lin Y, Zhao T, Tao S, Lai Z, Wei N, Yang R, Shao Y, He J (2019). miR-23b negatively regulates sepsis-Induced inflammatory responses by targeting ADAM10 in human THP-1 monocytes. Mediat Inflamm.

[27] Vincent JL, Moreno R, Takala J, Willatts S, De Mendonça A, Bruining H, Reinhart CK, Suter PM, Thijs LG (1996). The SOFA (sepsis-related organ failure assessment) score to describe organ dysfunction/failure. On behalf of the Working Group on Sepsis-Related problems of the European Society of Intensive Care Medicine. Intensive Care Med.

[28] Sungono V, Hariyanto H, Soesilo TEB, Adisasmita AC, Syarif S, Lukito AA, Widysanto A, Puspitasari V, Tampubolon OE, Sutrisna B, Sudaryo MK (2021). Cohort study of the APACHE II score and mortality for different types of intensive care unit patients. Postgrad Med J.

